# Aspects of the reproductive biology and gonad histology of Pagellus erythrinus (Actinopterygii: perciformes: Sparidae) in the Gulf of Antalya, NE Mediterranean Sea

**DOI:** 10.1007/s11259-025-10778-w

**Published:** 2025-05-30

**Authors:** Beytullah Ahmet Balci, Ramazan İkiz, Muhammed Nurullah Arslan

**Affiliations:** 1 Department of Aquaculture, Faculty of Fisheries, Akdeniz University, Antalya, Türkiye; 2 Department of Basic Sciences, Faculty of Fisheries, Akdeniz University, Antalya, Türkiye; 3 Department of Aquaculture, Faculty of Fisheries, Recep Tayyip Erdoğan University, Rize, Türkiye

**Keywords:** Gonad histology, *Pagellus erythrinus*, Protogynous hermaphrodite, Reproductive

## Abstract

This study examined the histological and anatomical changes in the gonads of the common pandora (*Pagellus erythrinus* L., 1758), a member of the Sparidae family, collected from the Gulf of Antalya (36° 49’ N, 30° 38’ E; 36° 46’ N, 31° 18’ E), NE Mediterranean Sea. A total of 273 specimens were sampled monthly from the region. The gonads were immediately dissected and fixed in 10% neutral buffered formaldehyde for 24–48 h, followed by Bouin’s solution for eight hours. They were then embedded in paraffin blocks after routine histological processing. Tissue Sect. (5 μm thick) were stained using hematoxylin-eosin and periodic acid-Schiff techniques for detailed analysis. As a protogynous hermaphrodite species, the common pandora exhibited four primary stages of gonadal development: the immature stage, female maturation stages (F1–F6), transitional stages (T1–T3), and male maturation stages (M1–M5). Based on these findings, it is recommended that fishing bans be enforced from late April to early September, coinciding with the species’ breeding season in the NE Mediterranean Sea.

## Introduction

The common pandora (*Pagellus erythrinus*), a member of the Sparidae family, is a commercially valuable species with a wide distribution across the eastern Atlantic Ocean from Norway to Angola, as well as the Mediterranean and Black Seas (Busalacchi et al. 2014; Lteif et al. 2020; Mejri et al. 2022; Rachedi et al. 2022). It is captured in Türkiye using different fishing methods, including trawls, longlines, gillnets, and trammel nets (Yapıcı and Filiz 2019). In the Gulf of Antalya, it holds significant economic importance due to its high market value, supporting small-scale fisheries and contributing to the local economy, while also showing potential as an alternative species for aquaculture development (Fostier et al. 2000; Klaoudatos et al. 2004; Rachedi et al. 2022).

Although it has an important place in handline fishing, it is a rarely caught species with a wide geographical distribution and a good market share (Valdés et al. 2004; D’Iglio et al. 2021; Mejri et al. 2022). However, despite being regarded as a promising alternative species for aquaculture alongside other members of the Sparidae family, limited research has been conducted on the aquaculture potential of the common pandora. (Cesaj et al. 1993; Güner et al. 2004).

For the aquaculture to be successful, its reproductive period, reproductive age, and especially reproductive physiology must be well known. For the breeding of different species in aquaculture, the determination of the genital structure, the gonad development, and other reproductive characteristics of the fish will provide more accurate results in production. Effective management of fisheries and fish farming requires a detailed understanding of the gonadal cycles of the common pandora and the underlying physiological mechanisms, such as hormonal regulation and environmental influences, to ensure sustainable production (Wootton and Smith 2014; Uribe et al. 2014; Ayyat et al. 2023). Therefore, the detailed histological structure and seasonal variations of teleostean gonads have garnered the attention of many investigators (Wootton and Smith 2014; Uribe et al. 2014; Ayyat et al. 2023). Thus, either through fishery or breeding methods, maximum efficiency will be achieved from the fish which will, in return, contribute to the national economy.

This study aims to investigate microscopically the anatomical and histological characteristics of the common pandora’s gonads, focusing on their cellular structure and developmental stages, to provide foundational data for its potential aquaculture production in the Gulf of Antalya.

## Materials and methods

**Fig. 1:**
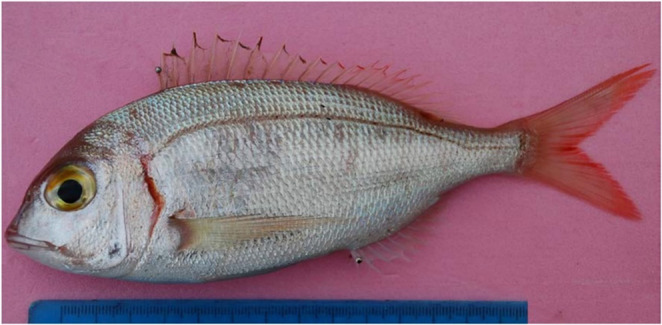
The common pandora (*Pagellus erythrinus*) from the Gulf of Antalya

**Fig. 2:**
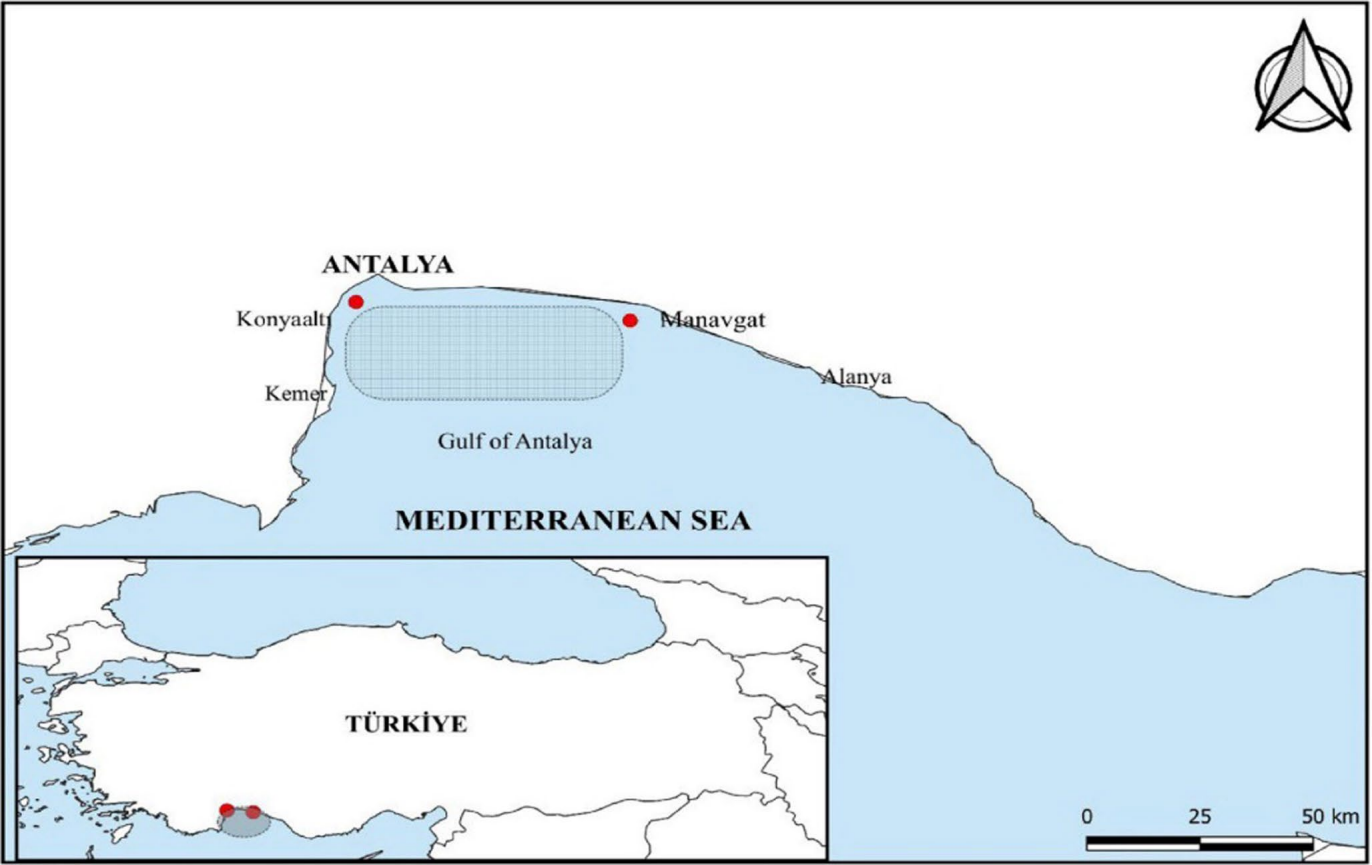
Map of the Gulf of Antalya showing the sampling area (36° 49’ N, 30° 38’ E to 36° 46’ N, 31° 18’ E) for *Pagellus erythrinus* (common pandora)

The samples were collected monthly throughout the year in favorable weather conditions (Fig. 1). A total of 273 fish were sampled, with 10 fish collected from each sampling area (36^o^ 49’ N, 30^o^ 38’ E; 36^o^ 46’ N, 31^o^ 18’ E) in the Gulf of Antalya every month (Fig. 2).

Using surgical fine-tipped scissors, a ventral incision was made on the fish’s body surface, starting an anterior to the anus and extending toward the operculum, taking care not to damage internal organs. Forceps were then used to remove the gonads by opening the abdominal cavity. Depending on the histochemical techniques to be applied, the removed gonads and pituitaries were fixed in Bouin’s solution for eight hours or in 10% neutral buffered formaldehyde solution for at least 24–48 h (Gabe 1976; Bancroft and Stevens 1977). After the tissues were fixed in the neutral formaldehyde, they were washed for one hour to eliminate the effect of the fixation fluid. The tissues kept in different concentrations of ethanol (30, 50, 70, 90, 100%) for a certain period (one hour in each) after the detection process was subjected to xylol and paraffin baths and blocked and made ready for cutting with a microtome. Sections of 5 µ thickness cut from the tissues blocked for routine histology were kept in an oven at 40 °C overnight. Each cut was stained according to Harris’s hematoxylin-eosin and Periodic Acid Schiff (PAS) staining procedures for the general histological examination (Gabe 1976; Bancroft and Stevens 1977). The slides stained and mounted in Canada balsam were examined under a binocular research microscope (Olympus CX21) and their microphotographs were taken for evaluation to identify the histological details (Gabe 1976; Bancroft and Stevens 1977; Roberts 1978; Curran 1995; Presnell et al. 1997). The opaque and dark bands of the scale and operculum of the fish were examined under a stereomicroscope for age estimation (Türkmen et al. 2010).

## Results

### Length, body mass, and age of the fish

It was determined that the ages, mass, and total length of 273 common pandora fish ranged from 0 to 8 ages, from 21.7 to 426.1 g and from 11.6 to 35.2 cm, respectively. Histological examination of the gonads revealed that the first developing sex was the female sex and then the male sex. Accordingly, it was observed that the fish were protogynous type hermaphrodites.

**Fig. 3:**
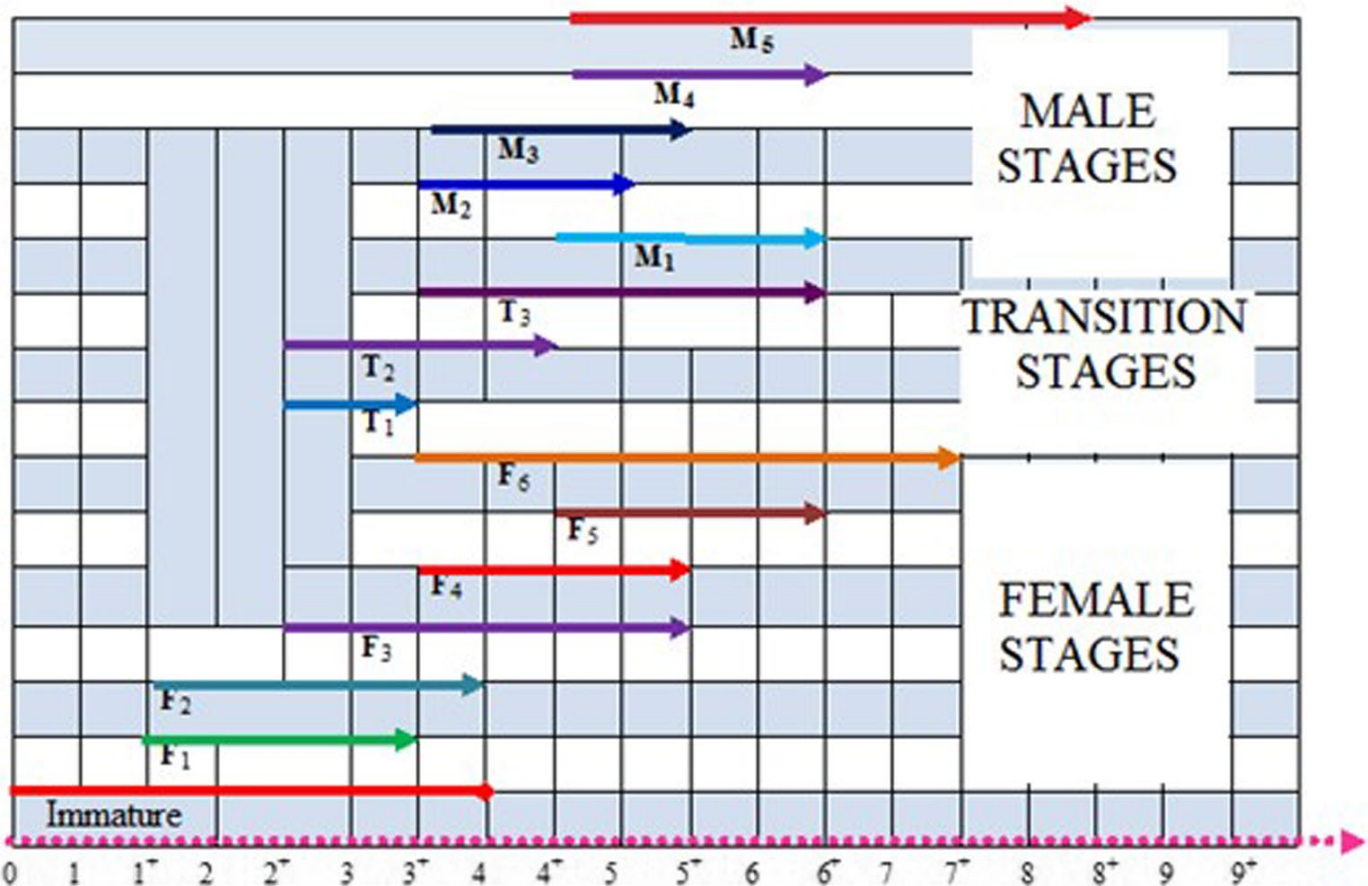
Gonadal development stage by age

This study revealed that the reproductive cycle of the common pandora (*Pagellus erythrinus*), a protogynous hermaphrodite, consists of four main stages: immature female, mature female, transitional phase (female-to-male sex reversal), and mature male (Fig. 3).

## Histological findings

### Immature

**Fig. 4:**
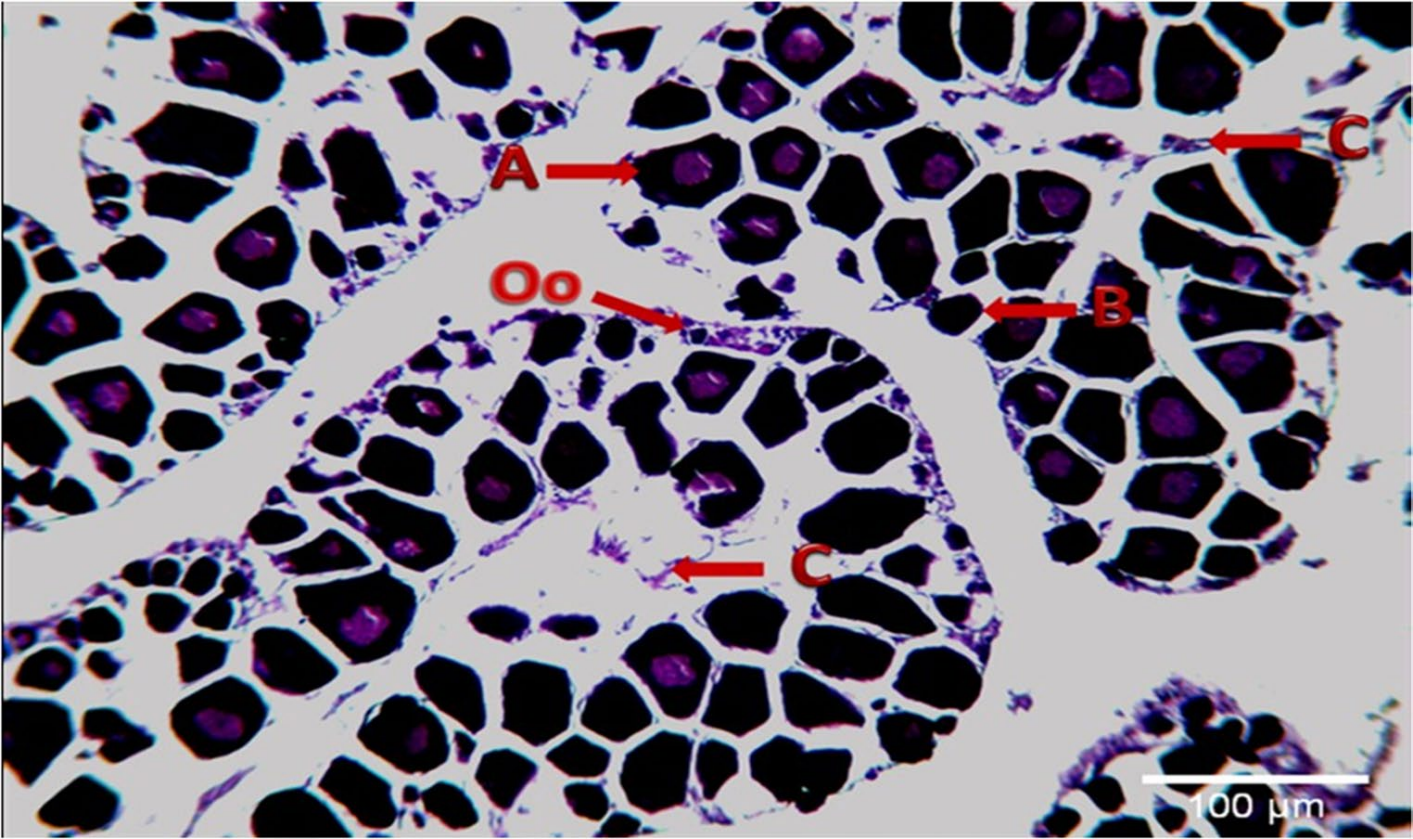
Immature oocytes (H + E) **A**: Perinuclear nucleoli **B**: The small oocytes dark colored **Oo**: Oogonium **C**: Ovarian folds (LT:16.02 cm, W:61.45)

At this stage, a rope-like, thin-veined appearance visible to the naked eye was observed in the gonads of the fish at I. age. It was detected that the granulation seen in the histological sections was not yet developed and that oocytes in the form of dark-colored packs were very close to each other shown in (Fig. 4). At the immature stage, a total of 42 fish at 0^+^-IV^+^ ages were caught. This stage was mostly observed between September and March. The highest rate (47.9%) was observed in November.

## Mature female

### Chromatin-nucleus stage (F1)

**Fig. 5:**
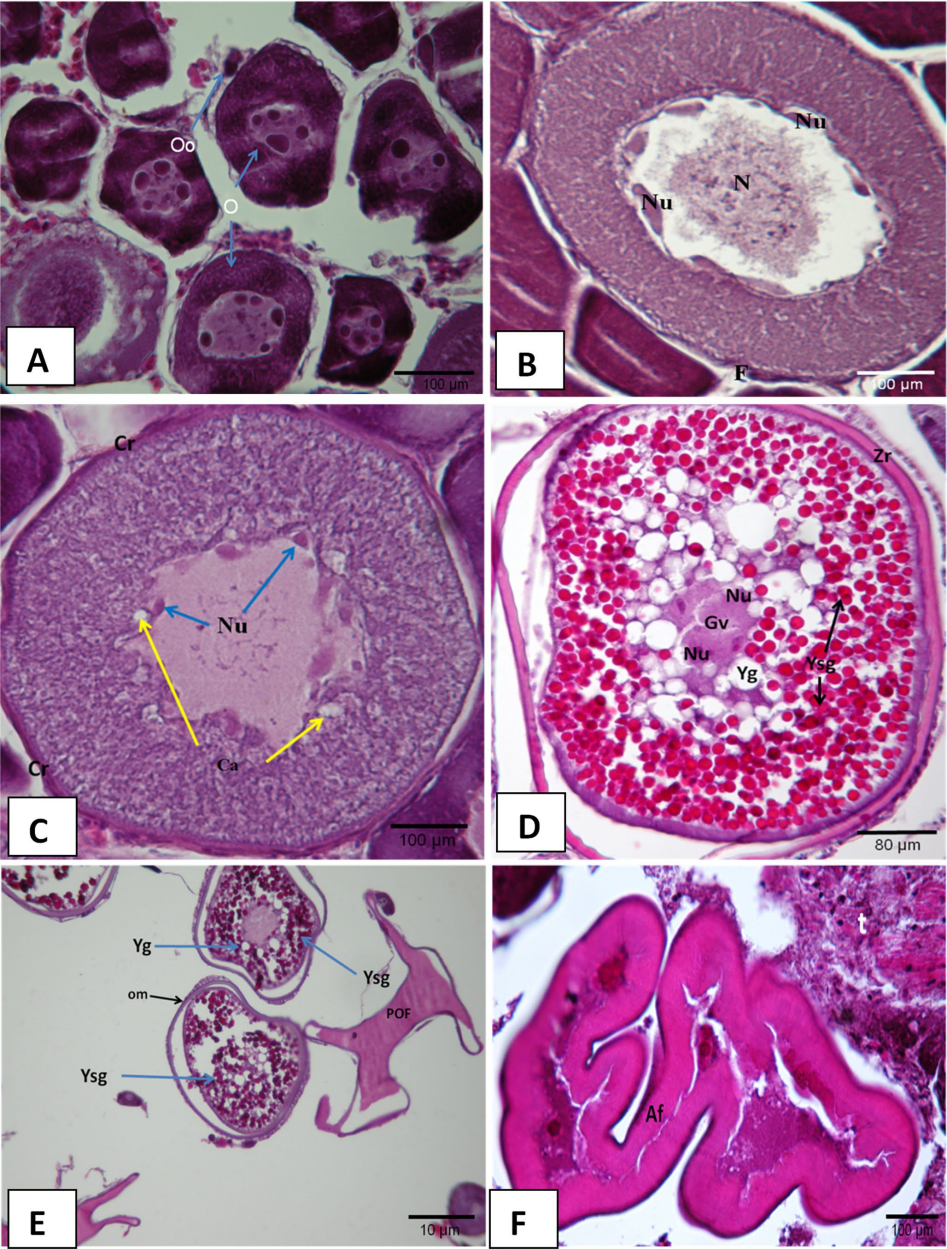
Mature stages of ovaries (H + E), **A**; (F1) **Oo**: Oogonium **O**:Oocyte (LT:16.65 cm, W:63.02 g) **B**; (F2) **N**:Nucleus **Nu**: Nucleolus **F**:Follicular cells (LT:18.51 cm, W:88.53 g) **C**; (F3) **Ca**: Cortical alveoli **Cr**:Corion (LT:20.18 cm, W:110.31 g) **D**; (F4) **Gv**:Germinal vesicle **Yg**:Yolk globules **Zr**:Zona radiata **Ysg**:Egg yolk granules (LT:18.47 cm, W:112.31 g) **E**; (F5) **POF**:Post ovulatory follicle **om**:Oocyte membrane thinned (LT:19.90 cm, W:117.39 g) **F**; (F6) **Af**:Atretic follicle **t**:Developing testicular tissue (LT:17.84 cm, W:79.74 g)

At the first phase of the meiotic prophase, oocytes and oogonia were observed. In this phase, the ovaries were yet to become functional, gaining a granular appearance. At the F1 phase, a total of 35 fish at I^+^-III^+^ ages were caught (Fig. 5A). Chromatin-nucleus stage was mostly observed between August and February. The highest rate (44.4%) was observed in January.

### Perinucleolus stage (F2)

Oocytes contain a homogeneous cytoplasm, a large nucleus, and many small peripheral nucleolus that look elongated. Compared to the previous stage, the nucleolus was more numerous and peripheral. In addition, more basophilic were observed in the cytoplasm compared to the previous stage. The developing oocytes were centrally located. In this study, a total of 10 fish at I^+^-IV^+^ ages were caught (Fig. 5B). Perinucleolus stage was mostly observed between January and April. The highest rate (27.8%) was observed in January.

### Cortical alveoli stage (F3)

Basophilic and inhomogeneous cytoplasm were monitored in the oocyte. Cortical alveoli that were not observed in the previous stage were detected in the cytoplasm. At the F3 stage, a total of 16 fish at II^+^-V^+^ ages were caught (Fig. 5C). This stage was mostly observed between February and July. The highest rate (20%) was observed in March.

### Vitellogenesis (F4)

Red-colored (acidophilic) yolk granules widely appear in the cytoplasm starting from the periphery of the cytoplasm. The oocytes increased in size compared to the previous stage and the oil droplets grew and became more pronounced. The growth of oocytes in size was found to be compatible with the accumulation of numerous nutrients observed in the cytoplasm. Zona radiata was thickened during the vitellogenesis stage. Germinal vesicles which were an indicator of oocyte maturation were monitored. At the F4 stage, a total of 7 fish at III^+^-V^+^ ages were caught (Fig. 5D). Vitellogenesis stage was mostly observed between March and June. The highest rate (5.7%) was observed in March.

### Maturity (F5)

It was observed that the migration of the germinal vesicle of the oocytes towards the animal pole was completed, that it lost its shape completely, and that the nucleolus completely disappeared in the cytoplasm. At the F5 stage, large hydrated oocytes and post-ovulatory follicles were observed. Oil droplets appeared larger by growing in number or merging. At a more advanced stage, all the lipid and protein content of the whole egg mingles and forms a homogeneous structure, and the chorion becomes thinner due to the growth of the egg in size. At the F5 stage, a total of 39 fish at IV^+^-VI^+^ ages were caught (Fig. 5E). Maturity stage was mostly observed between March and September. The highest rate (36.5%) was observed in May.

### After ovulation (F6)

Histological observations yielded many atretic follicles that were not removed from the ovary at the end of the breeding season. The presence of oocytes observed in the F4 and F5 stages was not observed in this stage. Male germ cells were observed to aggregate within female germ cells. Therefore, it was seen that it reached the final stage of the female character. At the F6 stage, a total of 35 fish at III^+^-VII^+^ ages were caught (Fig. 5F). This stage was mostly observed between April and December. The highest rate (37.4%) was observed in August.

In this study, the diameter of mature oocytes of common pandora (*Pagellus erythrinus*) ranged from 578 to 1100 μm, with an average of 839 ± 42.6 μm. The wide distribution of oocyte diameters indicated the presence of oocytes at different developmental stages during the reproductive period.

### Transition period of gonadal development

#### Spermatogonia clusters (T1)

**Fig. 6:**
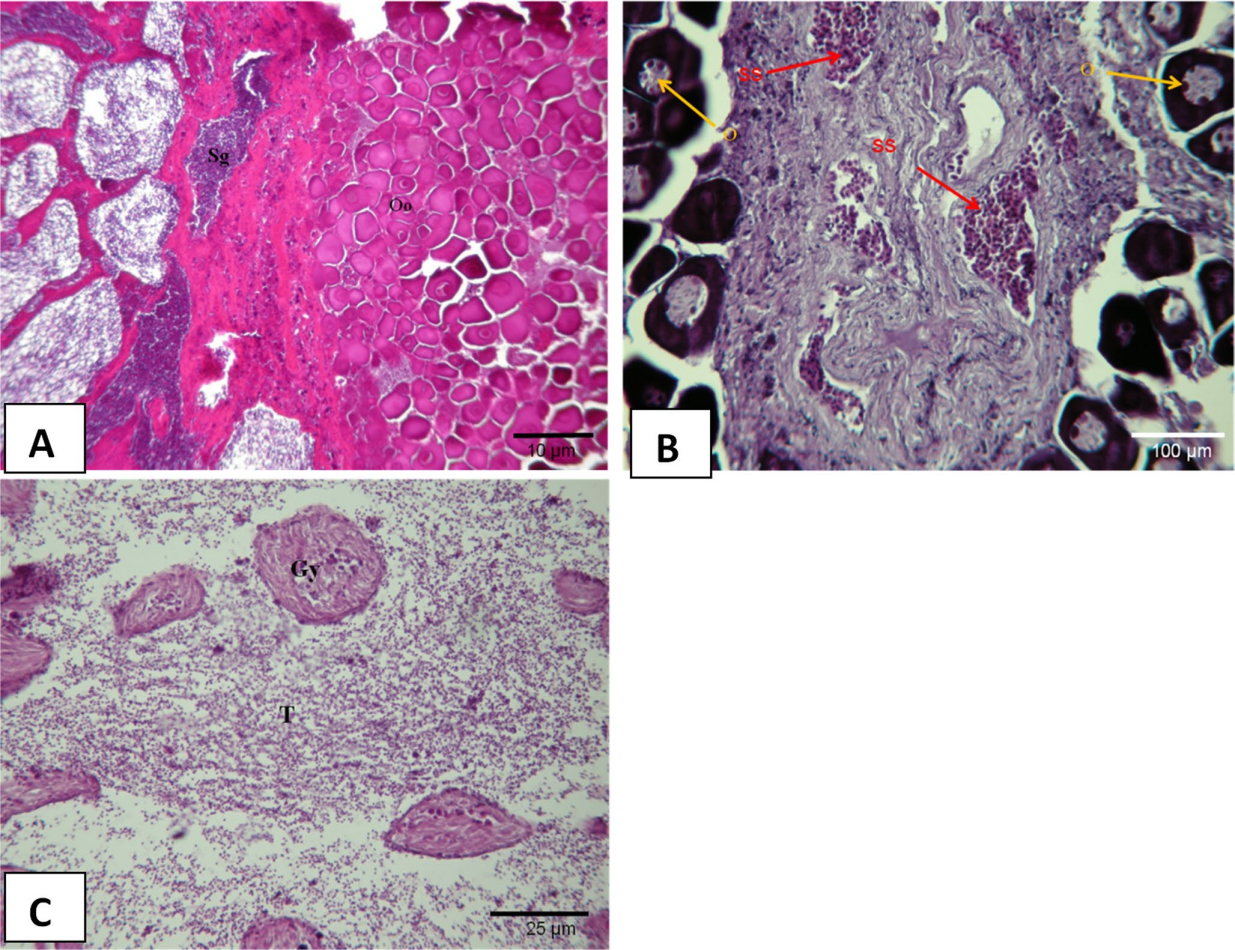
Transitional Gonadal Stages and Differentiations (H+E), **A**; (T1) **Oo: **Oogonia; **Sg: **Spermatogonia **B**; (T2) **SS:**Secondary spermatocyte **O: **Oocyte **C**; (T3) **Gy: **Regressed ovary **T: **Started to develop testicular

The first spermatogonia clusters were observed at the periphery of the ovary. For this investigation, a total of 10 fish at II^+^-III^+^ ages were caught (Fig. 6A). This stage was mostly observed between August and March. The highest rate (11.8%) was observed in October.

#### Transition stage (T2)

The presence of active spermatogenesis was observed at the periphery of an intact ovary. For this investigation, a total of 5 fish of II^+^-IV^+^ ages were caught (Fig. 6B). This stage was mostly observed between March and September. The highest rate (7.2%) was observed in August.

#### Final sex change (T3)

A severely regressed ovarian region and a nascent testicle were observed. At the T3 stage, a total of 7 fish at III^+^-VI^+^ ages were caught (Fig. 6C). The Final sex change stage was mostly observed between May and November. The highest rate (12.5%) was observed in August.

## Development of gonads in males

### Spermatogenesis (M1)

**Fig. 7:**
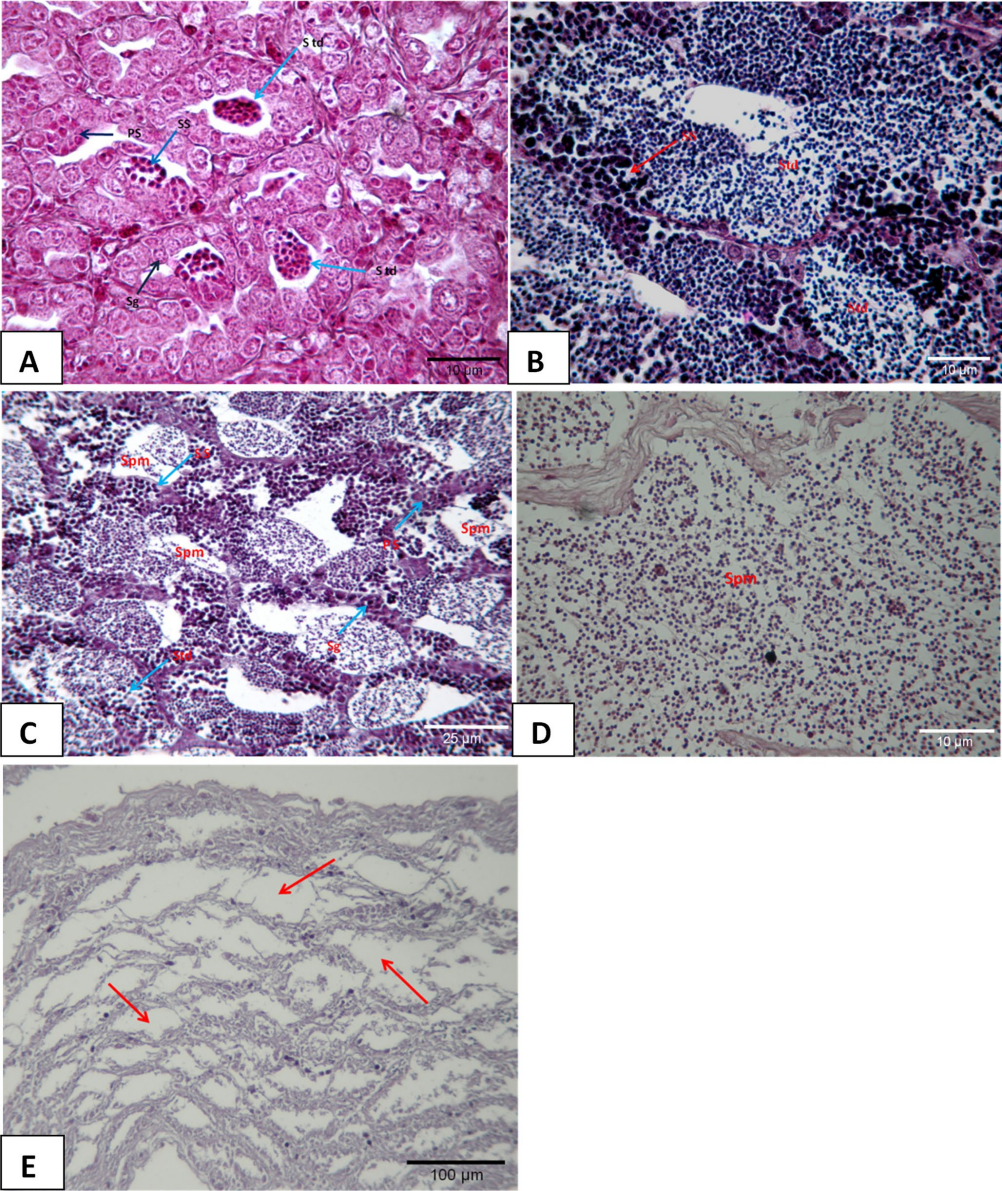
Male gonads and testicular differentiations (H+E) **A**; (M1) **Sg: **Spermatogonia **PS: **Primary spermatocyte **SS:**Secondary spermatocyte **Std: **Spermatid **B**; (M2) (PAS) **C**; (M3) **Spm: **Spermatozoa **D**; (M4) **E**; (M5) Testicular tubules **(arrow)**

Mostly spermatogonia and a number of primary spermatocytes were observed. A total of 13 fish in IV^+^-VI^+^ ages were caught (Fig. 7A). This stage was mostly observed between May and December. The highest rate (22.8%) was observed in December.

### Spermiogenesis (M2)

Spermatids and spermatozoa were observed more than spermatogenesis. However, sperm channels were not well developed. At the M2 stage, a total of 15 fish at III^+^-IV^+^ ages were caught (Fig. 7B). The Spermiogenesis stage was mostly observed between November and June. The highest rate (12.5%) was observed in February.

### Spermiation (M3)

The presence of plenty of spermatozoa together with spermatids was observed in the testicle and the deferent ducts. First, spermatogonium then primary and secondary spermatocytes, and then spermatid, and finally spermatozoa stages were reached in the lobules separated by thick connective tissue compartments. At the M3 stage, a total of 17 fish at III^+^-V^+^ ages were caught (Fig. 7C). Spermiation stage was mostly observed between February and July. The highest rate (20%) was observed between March and April.

### Sperm release (M4)

Following maturation, a well-developed central lumen and partially hollow but small number of spermatozoa were observed in the tubules in the cross-section of the testis. At this stage, a total of 14 fish at IV^+^-VI^+^ ages were caught (Fig. 6D). This stage was observed between March and September. The highest rate (16%) was observed in April.

### Recreation (M5)

After ovulation, regressed male gonad tissue was observed. There was no sperm production. Hollow tubules were observed. At this stage, a total of 8 fish at IV^+^-VIII^+^ ages were caught (Fig. 6E). This stage was observed between August and November. The highest rate (11.8%) was observed in October.

## Discussion

Common pandora (*P. erythrinus*) fish from the Sparidae family are protogynous hermaphrodites. Therefore, the development of the gonads as reported by other researchers is as follows; first, the development of females and then the transition period, and finally the development of male characterstics (Alekseev 1982; Devlin and Nagahama 2002; Valdés et al. 2004; Süzer and Kamacı 2005; Taşlı 2005).

The histological examinations of this study revealed that *P. erythrinus* exhibits asynchronous reproduction. It was determined that they mature at II age, show female characterstics between II and V ages, the transition from female to male between III and VI ages, and show male characterstics between IV and VIII ages. Valdés et al. (2004) reported that the lives of the Common pandora consist of four stages and these four stages were divided into sub-phases depending on the sexual maturity of the fish. This study also indicated that the four main stages reported in the previous studies and identified their sub-phases separately. These main stages were (I) Immature female, (II) female at the maturity stage, (III) Transition from female to male and (IV) Male. The first of these stages was the one in which sexual maturity had not yet been reached. At this stage, the histological sections removed from the gonads indicate that the short and closed ovarian folds were projected into the ovarian space. Early germ cells, oogonia, and small previtellogenic oocytes were observed along the axis and the edge which contain lamellas. These findings were supported by those of Valdés et al. (2004) and Elmajedeb and Alrwab (2023). The nascent immature ovary was found to have a granular structure. However, it was determined that the oocytes forming this granular structure were oocytes, oogonia, and previtellogenic oocytes which appeared to be packed close together. Fostier et al. (1987) and Kokokiris et al. (2001) studied the femaleness stage in five stages which are oogonia proliferation, previtellogenesis, vitellogenesis, final oocyte maturation, and ovulation. Glamuzina et al. (1998), and Valdés et al. (2004) divided the femaleness stage into two stages: immature female and female at maturity stage. Valdés et al. (2004) morphologically classified the mature female stage of the common pandora into six distinct sub-phases based on ovarian development. Similarly, the present study examined the female reproductive stages, focusing on histological changes, following the approach of Valdés et al. (2004).

Valdés et al. (2004) reported the presence of light-color-stained cytoplasms of oogonium and of the large nucleus at the chromatin nucleolus stage (stage F1). The oocyte was observed at the first stage of the oogonia and meiotic prophase. Ovaries that were yet to become functional are observed to gain a granular appearance at this stage (stage F1) and the presence of dark-stained oocytes was detected. Small previtellogenic oocytes were observed at the perinucleolus stage (stage F2) in which ovary folds are localized along the periphery. In addition, it was reported that a number of spermatogonia scattered along with the female gonadal tissue and melanophore macrophage centers were noted through the ovary folds (Marino et al. 2001). The oocytes of the fish examined in this study contained homogeneous cytoplasm, a large nucleus, and many small peripheral nucleolus that looked elongated. In addition, the cytoplasm was more basophilic than it was at the previous stages and centrally located as was the case for the developing oocytes at the F2 stage. McMillan (2007) and, Çakıcı and İşisağ Üçüncü (2007) reported that at the cortical alveoli stage (F3), the oocyte contains glycoprotein-structured cortical alveoli near the outer surface of the cytoplasm. This study also pointed out, as well as secondary oocyte growth at this stage, the presence of non-homogeneous cytoplasm which contains basophilic cortical alveoli which represents the F3 stage. Eosinophilic yolk granules, oocyte, and zona radiata in the cytoplasm thickened at this stage and the vitellogenesis stage (F4) was observed with the formation of vitellogenin in the oocytes as reported by Valdés et al. (2004). Water permeability in the oocytes which reached the F5 stage referred to as the maturity stage was observed and the vitellogenic oocytes at different developmental stages were among them. Cihangir (1996) and Valdés et al. (2004) reported that the sheath at this stage became quite thin and that it could explode with slight pressure. In present study revealed that water permeability in the oocytes occurred at this stage (F5 stage), and the thickness of the membranes that surrounded the oocyte at the initial stage of embryo development gradually decreased or broke. The oocytes at different developmental stages became dispersed throughout the membranes and male germ cells were present sporadically among these cells. In addition, the presence of large oocytes and post-ovulatory follicles was detected at stage F5. The lipid and protein content of some oocytes mingled and formed a homogeneous structure, the chorion became thinner due to the growth of the egg in size. The presence of atretic follicles after the reproductive stage of egg maturation (F6 stage) was reported by Valdés et al. (2004). It was also reported that most ovarian follicles regress in case of the death of follicular cells and oocytes, and those ovarian follicles were eliminated by phagocytic cells and atresia can be observed at all stages of follicle development (Junqueira et al. 1992). At stage F6 of the fish examined in this study, it appeared that the fish reached the final stage of the female character with the formation of eosinophilic cells as reposted by Valdés et al. (2004), and the presence of atretic follicles as reported by Junqueira et al. (1992). Also, the fact that male germ cells are more prevalent among these cells suggests that the fish will gain male sex characteristics.

Oocyte diameters are important for understanding common pandora’s reproductive biology (*P. erythrinus*) and making stock assessments. In this study, the diameter of mature oocytes ranged from 578 to 1100 μm, with an average of 839 ± 42.6 μm. Valdés et al. (2004) reported that they varied between 581.5 and 1008.7 μm, and Süzer and Kamacı (2005) reported that egg diameters were 802.01 ± 2.9 μm and that no difference was detected between egg sizes. These values ​​are similar to the egg diameter values ​​measured in the study.

These protogynous types of hermaphrodite fish are known to transition from female to male (Alekseev 1982; Devlin and Nagahama 2002; Valdés et al. 2004; Taşlı 2005; Süzer and Kamacı 2005). Protogynous-type hermaphroditism was observed in the histological sections of the samples examined. The female character of the fish at the early stages of their lives was observed to transition to the male character at later stages. The stage between the two sexes was referred to as the transitional stage and divided into three sub-stages which could also be distinguished morphologically. These stages are spermatogonia clusters (T1), transition stage (T2), and final sex change (T3) (Valdés et al. 2004). It was reported that the testicular tissue which develops at this stage becomes increasingly dominant (Marino et al. 2001). T1, T2, and T3 stages as stated by Valdés et al. (2004) were also identified separately in this study and the testicular tissue being more widely dispersed is in line with the findings of Valdés et al. (2004). As well as an increase in the interstitial tissue at the T1 stage, it was also observed that vitellogenic oocytes became eosinophilic, and the first spermatogonia clusters were formed at the periphery of the ovary. The presence of both germ cells in the gonads which shape the T2 stage of the transition stage was observed and no indication of any form of degeneration in ovaries was reported (Valdés et al. 2004). As reported by other researchers, both germ cells were observed together in the gonads examined in this study. The presence of active spermatogenesis became more common in the periphery of an intact ovary and the testicular tissue was found to be similar to the findings previously reported in this regard. T3 was the last stage of the transition stage. The formation of the testicular tissue became more pronounced however, ovarian tissue became degenerated (Johnson et al. 1998; Valdés et al. 2004). In this study, similar changes were observed in the histological structures of the gonads. Degenerate cells formed in clusters, the residues of the atretic egg cells and hollow spaces in the widely dispersed and well-developed testicular tissue pointed to the final stage of the transition stage as reported by Johnson et al. (1998) and Valdés et al. (2004).

Autopsy results showed that the testicles of the male fish were gray and composed of two equal lobes. These results were like the findings of Valdés et al. (2004). The sperm duct opened the posterior to the anus. In this study, the development of male gonads was discussed in five morphological stages as outlined in Fostier et al. (1987), Kokokiris et al. (2001), and Valdés et al. (2004). At different stages of development, basophilic stained male germ cells in the lobules of the testicular tissue at the spermatogenesis stage (M1) were like the findings of Valdés et al. 2004]. Mainly spermatogonia and a small number of primary spermatocytes are present. It is generally observed in October and December. Fostier et al. (1987), Kokokiris et al. (2001), and Valdés et al. (2004) reported the presence of spermatids and spermatocytes at spermiogenesis (M2). Spermatocytes and spermatozoa were observed in the testicular tissue at the spermiogenesis stage, and the highest rate was detected in March and April. It was also determined in this study that the sperm duct of the samples was not well developed although primary spermatocytes and a small number of spermatozoa were present. Many spermatozoa were detected in the deferent ducts and the testicles of the male gonads at the spermiation (M3) stage (Valdés et al. 2004). Many spermatozoa together with spermatids were observed in the testicles and the deferent ducts. First, spermatogonium, then primary and secondary spermatocytes, and then spermatid, and finally spermatozoa stages were detected in the lobules separated by thick connective tissue compartments in the testicular tissue. Spermiogenesis stages (M3) can be observed from March to July, however, the highest rate was observed between March and April. Although the tubules were mostly hollow in the testicular tissue at the sperm release (M4) stage, the presence of a small number of spermatozoa was reported occasionally (Valdés et al. 2004). In the hollow tubes after the sperm release, a small number of spermatozoa was observed with a well-developed central lumen in a part of the testicle. The testicular tissue of the male fish that reached the recreation stage (M5) indicated that there was no spermatozoa production anymore as reported by Valdés et al. (2004) and Kokokiris et al. (2001). Male tissue regressed with the end of the breeding season. This study also showed similar results.

## Conclusion

The gonad development of the protogynous type of hermaphrodite Common pandora fish was determined to be as follows, female, transition stage, and a male character. A total of 16 stages were determined: Immature stage, F1, F2, F3, F4, F5, F6 (femaleness stages), T1, T2, T3 (transition stages), and M1, M2, M3, M4, M5 (maleness stages). Common pandora which is one of the most valuable fish in the Mediterranean Sea should be allowed to reproduce at least once so that they can keep and sustain their population in balance with new generations. Therefore, fishery should be banned from the end of April to early September, their breeding season.

## Data Availability

No datasets were generated or analysed during the current study.
